# Case Report: Pituitary apoplexy temporally associated with acute alcohol intoxication, presenting as complete oculomotor nerve palsy in an SF-1 lineage gonadotroph PitNET

**DOI:** 10.3389/fendo.2026.1919724

**Published:** 2026-09-24

**Authors:** Xiang Tan, Yi Chang, Rile Wu, Yunqian Li

**Affiliations:** 1 Department of Neurosurgery, The First Hospital of Jilin University, Changchun, Jilin, China; 2 Department of Radiation Oncology, Jilin Provincial Cancer Hospital, Changchun, Jilin, China; 3 Department of Neurosurgery, Inner Mongolia People’s Hospital, Hohhot, Inner Mongolia, China

**Keywords:** alcohol, gonadotroph PitNET, hypopituitarism, oculomotor nerve palsy, PitNET, pituitary apoplexy, steroidogenic factor-1, transsphenoidal surgery

## Abstract

Pituitary apoplexy (PA) is an acute, potentially life-threatening neuroendocrine emergency caused by hemorrhage or infarction within the pituitary gland, most often arising in a previously unrecognized pituitary neuroendocrine tumor (PitNET). Reported precipitants include surgery, anticoagulation, dynamic pituitary testing, hypertension, and head trauma; alcohol is rarely implicated. We report a 61-year-old man with long-standing hypertension, type 2 diabetes mellitus and coronary artery disease (on aspirin after coronary stenting) who developed sudden thunderclap headache and complete right oculomotor nerve palsy the morning after consuming approximately 250 ml of distilled liquor (baijiu). Magnetic resonance imaging showed a 2.7 cm × 1.9 cm × 1.9 cm sellar mass with heterogeneous hemorrhagic signal, suprasellar extension and optic chiasm contact. Perioperative endocrine evaluation demonstrated secondary adrenal insufficiency, central hypothyroidism and low gonadotropins, consistent with multi-axis hypopituitarism. After aspirin withdrawal and immediate parenteral hydrocortisone, the patient underwent endoscopic endonasal transsphenoidal resection on day 8. Histopathology confirmed an SF-1 lineage gonadotroph PitNET with extensive infarction (Ki-67 2%; FSH+, SF-1+, PIT-1-, T-PIT-). At 3 months, the right oculomotor palsy had partially recovered, with improvement in ptosis and ocular motility but incomplete resolution. Follow-up contrast-enhanced magnetic resonance imaging showed no residual or recurrent tumor, a decompressed optic chiasm and a midline pituitary stalk, and the markedly elevated presenting 24h urinary free cortisol had returned to the laboratory reference range. Anterior pituitary function nonetheless remained deficient across the corticotroph, thyrotroph, and gonadotroph axes, and the patient continued stable hydrocortisone and levothyroxine replacement. This case adds to the limited literature on PA temporally associated with alcohol intake, illustrates the value of World Health Organization 2022 lineage-based PitNET classification, and supports individualized, semi-elective surgery in haemodynamically stable patients without acute visual-pathway compromise.

## 1 Introduction

Pituitary apoplexy (PA) is an acute clinical syndrome resulting from hemorrhage and/or infarction of the pituitary gland, usually within a pre-existing pituitary neuroendocrine tumor (PitNET) (1, 2). It is a recognized endocrine emergency because abrupt destruction of corticotroph function can precipitate life-threatening secondary adrenal insufficiency, while expansion of the sellar contents may compress the optic apparatus and the cavernous sinus cranial nerves (3, 4). Symptomatic apoplexy is reported in approximately 2%–12% of pituitary adenomas, and a majority of patients have no prior diagnosis of a sellar lesion at presentation (1, 2).

A range of precipitating factors has been described, including major surgery, anticoagulant or antiplatelet therapy, dynamic pituitary function testing, dopamine agonists, head trauma, pregnancy, and systemic conditions such as hypertension and diabetes mellitus (3, 4). Alcohol consumption, by contrast, is only sporadically reported as a trigger, and the mechanistic basis remains poorly defined. Here, we describe a PA occurring after acute alcohol intoxication and presenting with isolated complete oculomotor nerve palsy in a patient with multiple cardiovascular risk factors, in whom histopathology confirmed an SF-1 lineage gonadotroph PitNET. We integrate 3-month clinical, endocrine, and imaging follow-up and discuss the proposed neuroendocrine and hemodynamic mechanisms, the differential recovery of cranial nerve palsy, and the rationale for individualized surgical timing. The report follows the CARE (CAse REport) guidelines (5).

## 2 Case description

### 2.1 Patient information and presentation

A 61-year-old man presented to the emergency department with sudden, severe throbbing headache and right eyelid drooping. The symptoms began on awakening, the morning after he had consumed approximately 250 ml of distilled liquor (baijiu, ~52% alcohol by volume) on a single evening and became intoxicated. He reported a 15-year history of regular social alcohol use. His medical history included hypertension of 15 years (treated with nifedipine and metoprolol; usual blood pressure ~120/80 mmHg), type 2 diabetes mellitus (metformin and dulaglutide; fasting glucose ~7 mmol/L), and coronary artery disease treated by percutaneous coronary intervention with stent placement in 2012. He had taken aspirin continuously until the day of symptom onset.

He had no history of cigarette smoking. His family history was notable for hypertension in his father, with no known family history of pituitary or other endocrine tumors. Apart from the episode of acute alcohol consumption described above, no other remarkable occupational or psychosocial factors were identified.

### 2.2 Clinical and ophthalmological findings

On admission the patient was alert and haemodynamically stable (blood pressure 125/82 mmHg, heart rate 78 beats/min). Neuro-ophthalmological examination revealed complete right oculomotor (cranial nerve III) palsy: severe ptosis, a fixed dilated right pupil (5.5 mm) with absent direct and consensual light reflex, and restricted adduction, elevation and depression. Best-corrected visual acuity was 0.4 (right) and 1.0 (left), and formal visual field testing showed no defect. Ophthalmological assessment considered mild lenticular opacity (early cataract) the most likely explanation for the reduced right visual acuity, although a contribution from acute sellar pathology could not be completely excluded; the absence of any visual-field deficit argued against significant compression of the anterior visual pathway. Ophthalmology favored oculomotor nerve palsy as the principal abnormality. The remaining cranial nerves were intact and there were no signs of meningism.

A detailed cranial-nerve examination confirmed that, apart from the right oculomotor nerve, the other cranial nerves were intact: visual acuity and visual fields were preserved (cranial nerve II), the trochlear (IV) and abducens (VI) nerves were unaffected, facial sensation was normal (V), the facial nerve (VII) was normal, and there was no lower cranial-nerve deficit. General systemic examination was consistent with the patient’s known comorbidities. The cardiovascular history included hypertension, type 2 diabetes mellitus and prior coronary stenting; resting electrocardiography showed sinus rhythm with changes consistent with an old anterior myocardial infarction, and echocardiography demonstrated normal left-ventricular global longitudinal strain with essentially synchronous wall motion. This was an asymptomatic, pre-existing and clinically stable finding, and no acute cardiac event was identified during admission. The respiratory examination was unremarkable, with clear breath sounds bilaterally and no respiratory distress. No standard optical coherence tomography (OCT) of the retinal nerve fiber layer was performed; because the deficit was an efferent oculomotor and pupillomotor palsy rather than an afferent optic-nerve lesion, and the visual field was normal, OCT was considered unlikely to add diagnostic value or alter management, and a dilated fundus examination was unremarkable.

### 2.3 Endocrine and laboratory evaluation

Baseline anterior pituitary testing was consistent with hypopituitarism (**Table 1**). The 08:00 serum cortisol was markedly low at 63.33 nmol/L (reference 185–624), indicating secondary adrenal insufficiency, with low gonadotropins (FSH 1.26 mIU/ml, LH 1.57 mIU/ml), low-normal prolactin and low growth hormone. Thyroid function was not assessed at the referring hospital at presentation and was characterized postoperatively (see below and **Table 1**). Notably, a 24h urinary free cortisol obtained at presentation was elevated (14,693.58 nmol/24h; reference 108–961) in the context of a high 24h urine volume (5.5 L) and concurrent hyperglycaemia. Importantly, this urine collection was completed before the initiation of any exogenous glucocorticoid therapy, excluding contamination or assay cross-reactivity from administered hydrocortisone as an explanation. We interpret this dissociation cautiously. Although an acute stress-related surge in adrenal cortisol secretion at the onset of apoplexy, glucose-driven osmotic diuresis increasing urinary steroid clearance, and the analytical limitations of urinary free cortisol assays at extreme values may all have contributed, the precise explanation for this marked elevation remains uncertain. Because plasma adrenocorticotropic hormone (ACTH), late-night salivary cortisol, dexamethasone suppression testing and a repeat preoperative urinary free cortisol were unavailable, transient hypercortisolism cannot be completely excluded; these are acknowledged limitations. The subsequent return of the value into the reference range was observed while the patient was receiving glucocorticoid replacement, which itself suppresses endogenous cortisol output and therefore limits the inference that can be drawn from this normalization; nonetheless, the absence of any clinical features of Cushing syndrome throughout follow-up makes persistent endogenous hypercortisolism unlikely.

**Table 1:** Anterior pituitary hormonal profile at presentation, postoperative day 2, and 3-month follow-up.

Parameter (unit)	Reference range	At presentation	POD 2	3 months
Cortisol, 08:00 (nmol/L)	185-624	63.33	81.38	65.40
Cortisol, 16:00 (nmol/L)	—	79.82	681.39	130.36
Cortisol, 24:00 (nmol/L)	< 276	86.98	13.59	12.33
TSH (µIU/ml)	0.35–4.94	—	0.162	0.298
Free T4 (pmol/L)	9.01–19.05	—	14.21	7.58
Free T3 (pmol/L)	2.43–6.01	—	2.11	2.87
FSH (mIU/ml)	0.9–10.9	1.26	1.04	0.84
LH (mIU/ml)	2.80–6.80	1.57	0.42	< 0.20
Prolactin (ng/ml)	2.64–13.13	3.13	3.38	11.79
GH (ng/ml)	0.02–1.23	0.68	0.11	< 0.05
24h urine free cortisol (nmol/24h)	108–961	14,693.58	6273.72	605.57
24h urine volume (L)	—	5.5	4.0	3.5

TSH, thyroid-stimulating hormone; FSH, follicle-stimulating hormone; LH, luteinizing hormone; GH, growth hormone; POD, postoperative day. Dashes indicate parameters not measured at that time point; thyroid function (TSH, free T4, free T3) was not assessed at the referring hospital at presentation. The presenting 24h urinary free cortisol was collected before the initiation of any exogenous glucocorticoid therapy. Values are reported as provided by the performing laboratory; reference ranges follow that laboratory. A reference range for midnight (24:00) cortisol is not provided. From the early postoperative period onward the patient was receiving glucocorticoid replacement (hydrocortisone 15 mg in the morning and 10 mg in the evening) and levothyroxine 12.5 µg daily; postoperative cortisol values therefore reflect exogenous replacement and the timing of sampling relative to dosing rather than endogenous secretion (e.g., the postoperative day 2 midnight value was obtained shortly after an evening hydrocortisone dose, whereas the 16:00 value represents a pre-dose trough).

### 2.4 Imaging

Contrast-enhanced sellar magnetic resonance imaging (MRI) demonstrated an enlarged sella containing a well-defined mass measuring 2.7 cm × 1.9 cm × 1.9 cm with heterogeneous mixed T1 and T2 signal consistent with intratumoral hemorrhage. The mass extended into the suprasellar cistern with upward displacement of the pituitary stalk and contact with the optic chiasm, and showed heterogeneous mild enhancement. The right margin abutted the right cavernous sinus with an indistinct interface (**Figures 1A, B**). These appearances were radiologically consistent with hemorrhagic PA.

**Figure 1:**
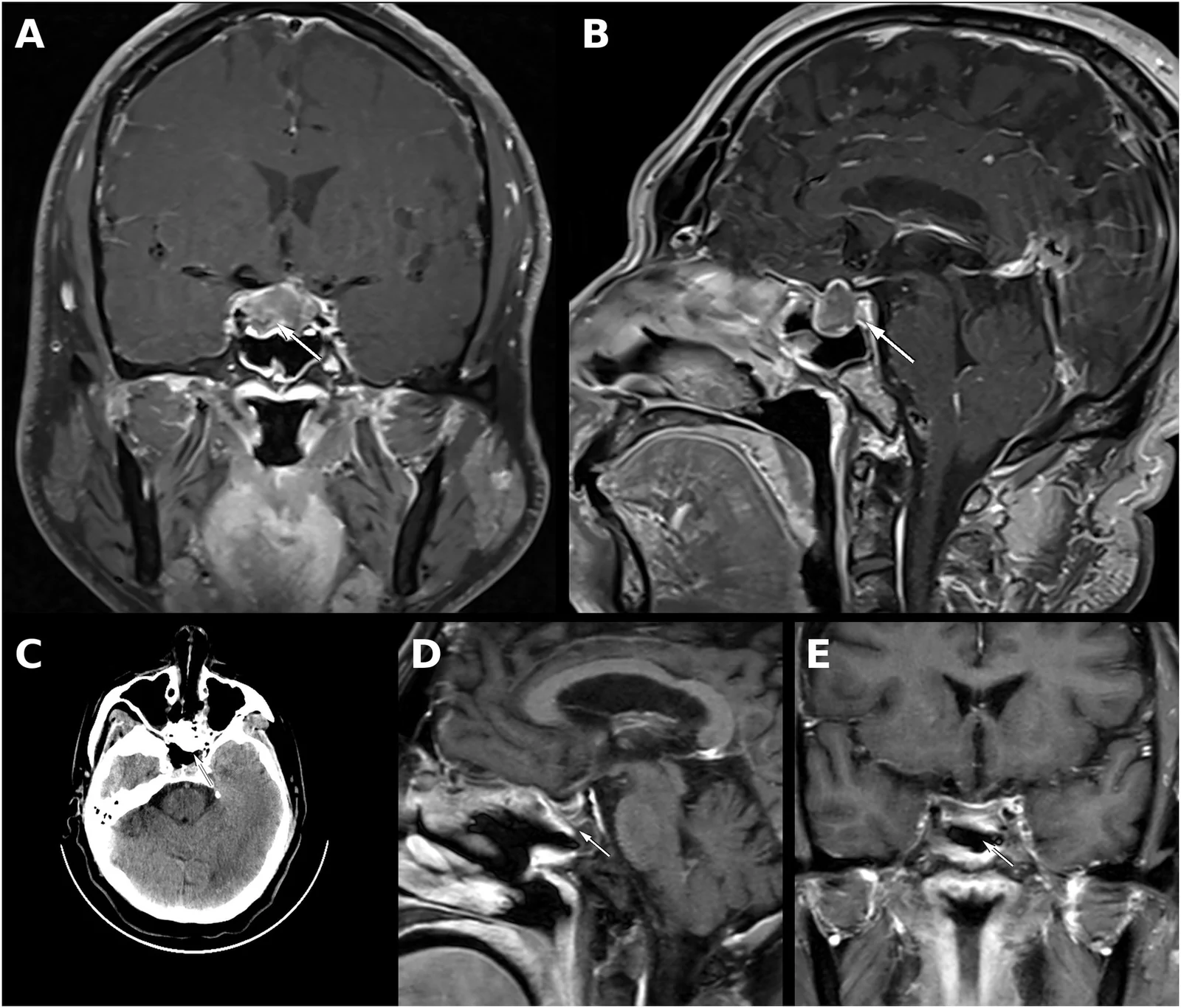
Preoperative and postoperative imaging. **(A)** Preoperative coronal T1-weighted contrast-enhanced image showing a 2.7 cm × 1.9 cm × 1.9 cm sellar mass with heterogeneous signal consistent with intratumoral hemorrhage (arrow), suprasellar extension and contact with the optic chiasm; the right margin abuts the right cavernous sinus. **(B)** Preoperative sagittal T1-weighted image showing the same mass (arrow) with heterogeneous, peripherally enhancing signal, suprasellar extension and upward displacement of the pituitary stalk. **(C)** Early postoperative axial computed tomography showing the transsphenoidal corridor with an opened sphenoid sinus (arrow) and low-density change in the sellar region indicating adequate decompression, without hemorrhage, pneumocephalus, or cerebrospinal fluid leak. **(D, E)** Three-month postoperative contrast-enhanced T1-weighted MRI in the sagittal **(D)** and coronal **(E)** planes. Expected post-transsphenoidal changes are seen, with a thin residual pituitary gland apposed to the floor of the sella (arrows) and no abnormal enhancement to indicate residual or recurrent tumor. The pituitary stalk is largely midline, and the optic chiasm, cavernous sinuses and both internal carotid arteries are unremarkable, consistent with durable decompression of the optic apparatus.

### 2.5 Therapeutic intervention

Because the patient was alert, haemodynamically stable, and had full visual fields without clinically significant optic-pathway compromise, an isolated oculomotor palsy that did not by itself mandate emergency decompression, and required time for antiplatelet washout before surgery, a strategy of immediate medical stabilization followed by semi-elective surgery was selected after multidisciplinary discussion. The decision to withhold aspirin was made by the neurosurgical team weighing the hemorrhagic context of the apoplexy against thrombotic risk, noting that coronary stenting had been performed more than 10 years previously. Aspirin was withheld and parenteral stress-dose hydrocortisone (100 mg 3 times daily) was commenced before confirmatory endocrine results, in keeping with the management of suspected acute corticotroph failure. The patient remained neurologically and ophthalmologically stable throughout the observation period, without deterioration of visual acuity, visual fields or consciousness, which supported a planned rather than emergency operation. Eight days after aspirin withdrawal, the patient underwent endoscopic endonasal transsphenoidal resection. Intraoperatively, hemorrhagic, necrotic tumor was identified and was partially adherent to the right cavernous sinus wall; gross total resection was achieved and the sellar floor was reconstructed.

### 2.6 Histopathology

Histological examination showed fragmented tan-brown tissue measuring 2.8 cm × 2.5 cm × 0.3 cm in aggregate. Microscopically, the greater part of the specimen was infarcted, with pale eosinophilic ghost areas in which nuclear detail was lost, alternating with smaller foci of preserved viable tumor (**Figure 2A**). In the preserved areas, the tumor was composed of a monomorphic population of cells with eosinophilic cytoplasm and small round nuclei arranged in nests and trabeculae separated by a delicate vascular stroma; nuclear atypia and mitotic figures were not identified (**Figures 2B, C**). Immunohistochemistry was positive for FSH, steroidogenic factor-1 (SF-1), synaptophysin, chromogranin A and CAM5.2, with a Ki-67 labelling index of 2% and p53 expression in approximately 10% of nuclei, and negative for GH, PRL, ACTH, LH, TSH, PIT-1, T-PIT and estrogen receptor (**Figures 2D–H**). GATA3 staining was not available at our institution; the diagnosis of an SF-1 lineage gonadotroph PitNET was therefore established on the basis of diffuse nuclear SF-1 positivity together with FSH expression and the absence of PIT-1 and T-PIT, in accordance with the World Health Organization (WHO) 2022 classification of pituitary tumors (6). The extensive infarction confirms a predominantly infarctive rather than purely hemorrhagic apoplexy.

**Figure 2:**
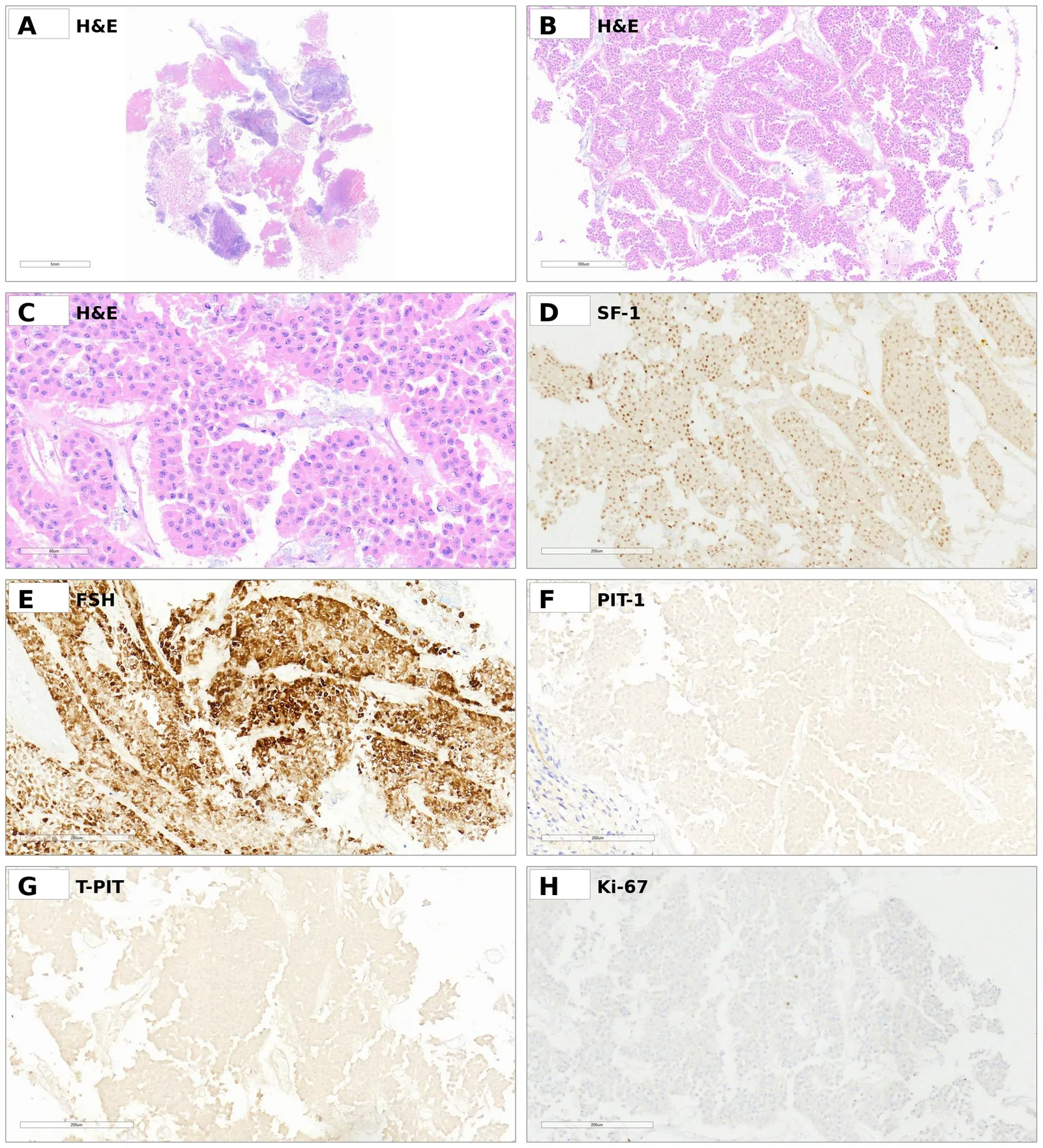
Histopathological and immunohistochemical features of the resected sellar lesion. **(A)** Haematoxylin and eosin (H&E) staining of the whole specimen shows that the greater part of the submitted tissue is infarcted, with pale eosinophilic ghost areas alternating with smaller foci of preserved, basophilic viable tumor. **(B)** H&E staining of a preserved area shows tumor cells arranged in nests and trabeculae separated by a delicate vascular stroma. **(C)** H&E staining at higher magnification shows a monomorphic population of cells with eosinophilic cytoplasm and small round nuclei; nuclear atypia and mitotic figures are not identified. **(D)** Steroidogenic factor-1 (SF-1) shows diffuse nuclear positivity. **(E)** Follicle-stimulating hormone (FSH) shows cytoplasmic positivity. **(F)** PIT-1 is negative. **(G)** T-PIT is negative. **(H)** Ki-67 shows a low labelling index of approximately 2%. Original magnifications: **(A)** ×5, **(B)** ×100, **(C)** ×400, **(D–H)** ×200. Scale bars: **(A)** 5 mm, **(B)** 300 µm, **(C)** 60 µm, and **(D–H)** 200 µm.

### 2.7 Early postoperative course

Recovery was uneventful, without cerebrospinal fluid leak, postoperative hemorrhage or new neurological deficit. Early postoperative computed tomography confirmed the transsphenoidal corridor and adequate sellar decompression (**Figure 1C**). Stress-dose glucocorticoids were tapered to physiological replacement. Postoperative day 2 testing confirmed persistent central hypoadrenalism, central hypothyroidism and hypogonadism (08:00 cortisol 81.38 nmol/L; TSH 0.162 µIU/ml with free thyroxine 14.21 pmol/L; FSH 1.04 mIU/ml; LH 0.42 mIU/ml), indicating a continued need for replacement therapy. He was discharged on postoperative day 7 on oral hydrocortisone (15 mg morning, 10 mg evening) and levothyroxine, the latter initiated conservatively at 12.5 µg daily in view of his advanced age and cardiovascular comorbidity (coronary artery disease with prior stenting), with planned up-titration under endocrine supervision. The right oculomotor palsy persisted at discharge. Structured follow-up was arranged in endocrinology (hormonal review) and neurosurgery (clinical and MRI surveillance). The overall clinical course is summarized in **Figure 3**.

**Figure 3:**
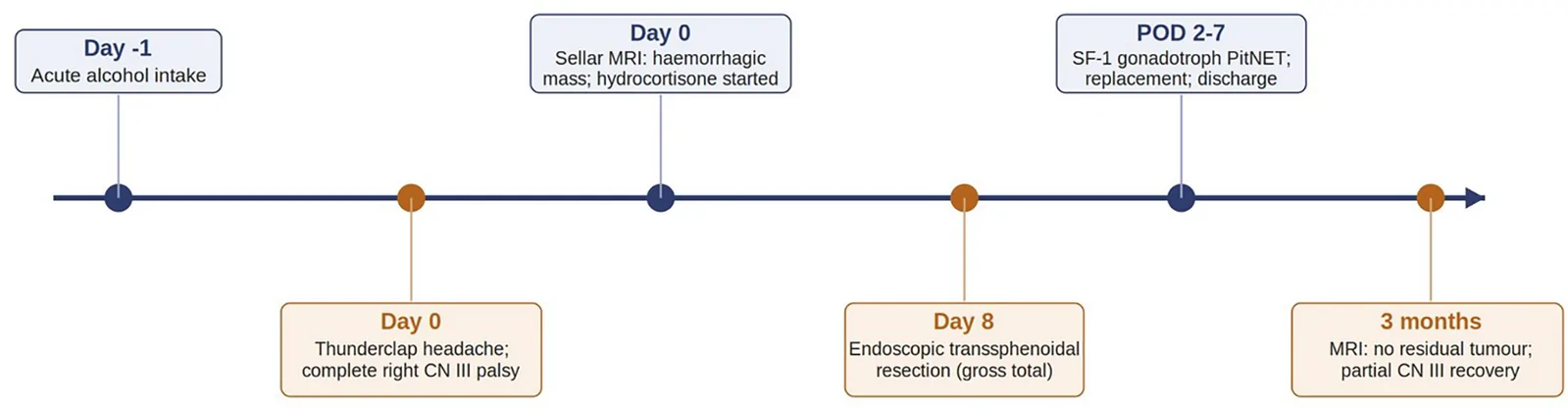
Timeline of the clinical course, from acute alcohol intake (day -1) and symptom onset with complete right oculomotor nerve palsy (day 0), through diagnostic imaging and initiation of glucocorticoid cover (day 0), endoscopic transsphenoidal resection (day 8), histopathological diagnosis and hospital discharge (postoperative days 2–7), to the 3-month follow-up demonstrating no residual tumor and partial recovery of the oculomotor palsy. CN III, oculomotor nerve; PitNET, pituitary neuroendocrine tumor; POD, postoperative day; SF-1, steroidogenic factor-1.

## 3 Three-month follow-up

The patient was reviewed approximately 3 months after surgery.

Neuro-ophthalmological outcome. The right oculomotor nerve palsy had partially recovered. Compared with the complete palsy at presentation, the degree of ptosis was reduced and extraocular movements had partly improved, although recovery remained incomplete with residual deficit. This pattern of gradual, partial recovery is consistent with the natural course of third-nerve palsy after PA, in which improvement typically occurs over weeks to several months.
Endocrine outcome. Anterior pituitary function remained deficient across multiple axes (**Table 1**). The 08:00 serum cortisol was low (65.40 nmol/L; reference 185–624) and the midnight cortisol was 130.36 nmol/L, indicating persistent secondary adrenal insufficiency requiring continued glucocorticoid replacement. Thyroid function showed a low free thyroxine (7.58 pmol/L; reference 9.01–19.05) with low free triiodothyronine (2.87 pmol/L) and low thyroid-stimulating hormone (0.298 µIU/ml), consistent with central hypothyroidism; the subnormal free thyroxine on a low fixed levothyroxine dose of 12.5 µg daily indicates that replacement was insufficient; the patient was therefore advised to undergo stepwise up-titration of levothyroxine at endocrine follow-up, titrated to a free thyroxine level in the mid-to-upper reference range rather than to thyroid-stimulating hormone. Gonadotropins remained low (FSH 0.84 mIU/ml; LH <0.20 mIU/ml), and low growth hormone (<0.05 ng/ml) and prolactin concentrations were also observed, in keeping with persistent multi-axis hypopituitarism. Because dynamic testing and Insulin-like growth factor 1 (IGF-1) were not performed, the random growth hormone value is descriptive only and is not interpreted as confirming growth hormone deficiency. Notably, the 24h urinary free cortisol, which had been markedly elevated at presentation, had fallen within the laboratory reference range (605.57 nmol/24h; reference 108–961) with a reduced 24h urine volume (3.5 L), although interpretation is limited by concurrent glucocorticoid replacement. The patient continued hydrocortisone (15 mg in the morning, 10 mg in the evening) and levothyroxine (12.5 µg daily).
Imaging outcome. Follow-up contrast-enhanced MRI at 3 months demonstrated expected post-surgical changes, including an enlarged sella with a depressed and partially discontinuous sellar floor consistent with the transsphenoidal approach. The residual pituitary gland appeared thin and apposed to the sellar floor, with no abnormal enhancement to suggest residual or recurrent tumor. The pituitary stalk had returned to a largely midline position, and the optic chiasm, both internal carotid arteries and the cavernous sinuses were unremarkable, confirming durable decompression of the optic apparatus (**Figures 1D, E**).
Functional status. The patient remained clinically well, adherent to hormone replacement, and reported no new symptoms or adverse events during the follow-up interval. Ongoing endocrine and neurosurgical surveillance was arranged, including levothyroxine dose optimization and continued imaging follow-up.

## 4 Discussion

### 4.1 Current understanding of pituitary apoplexy

PA reflects an acute imbalance between the metabolic demand of a sellar tumor and its vascular supply. PitNETs are perfused largely by a fragile, often incomplete arterial network rather than the hypophyseal portal system that supplies the normal anterior lobe, and rapid tumoral growth can outstrip this supply (7, 8). Compression of the superior hypophyseal vessels and of the gland against the diaphragma sellae further predisposes to ischaemia and secondary hemorrhage (8). Clinically, PA is defined by the abrupt onset of headache, variably accompanied by visual loss, ophthalmoplegia, altered consciousness and acute hypopituitarism (1, 3). Contemporary management is multidisciplinary and risk-stratified; corticosteroid cover for presumed corticotroph failure is the single most important early intervention, while the decision between surgery and conservative care is individualized (3, 9).

### 4.2 Differential diagnosis and the challenge of apoplexy mimicking stroke

The acute presentation of severe headache with a complete unilateral oculomotor nerve palsy has a broad differential that the sellar diagnosis must be distinguished from. A posterior communicating artery aneurysm is the classic, must-exclude cause of a painful pupil-involving third-nerve palsy and was excluded here by neuro-imaging. Microvascular or ischaemic oculomotor palsy related to this patient’s hypertension and type 2 diabetes characteristically spares the pupil and would not account for the sellar mass. Cavernous sinus pathology (thrombosis, inflammation or tumor invasion), other sellar and parasellar lesions (craniopharyngioma, Rathke cleft cyst, hypophysitis or metastasis), and ophthalmoplegic migraine or an orbital apex syndrome were likewise considered and rendered unlikely by the clinical picture and the characteristic hemorrhagic sellar mass on MRI. Importantly, because acute apoplexy frequently presents with focal neurological deficits and thunderclap headache, it is readily mistaken for an acute cerebrovascular event, especially when the first investigation is non-contrast CT. Gaydarski et al. described a patient initially transferred for endovascular treatment of a presumed hemorrhagic stroke in whom MRI subsequently revealed a pituitary macroadenoma with apoplexy (10). Our case reinforces the need for a low threshold for dedicated pituitary MRI and for early multidisciplinary coordination when headache is accompanied by ophthalmoplegia, so that apoplexy is not overlooked in the emergency setting.

### 4.3 Potential mechanisms linking alcohol consumption and pituitary apoplexy

Alcohol is an uncommon and incompletely understood precipitant of PA, and causation in any single case cannot be established; indeed, in contrast to the atypical triggers summarized in **Supplementary Table S1** (18–21), a discrete drinking episode is rarely documented as a precipitant in the English-language literature. In the present patient several biologically plausible contributors coincided. Acute alcohol intake produces blood-pressure variability and sympathetic activation, and both acute hypertensive surges and hypotension have been linked to apoplexy (11). Ethanol also has recognized effects on platelet function and coagulation and on vascular tone (12), and chronic alcohol exposure perturbs hypothalamic-pituitary regulation (13). Superimposed on pre-existing hypertension, diabetic microvascular disease and coronary atherosclerosis, these transient hemodynamic and rheological changes could plausibly tip an already marginal tumoral circulation toward infarction. We therefore frame alcohol as a possible contributory trigger acting on a vulnerable substrate, rather than as an independently proven cause, and note that the temporal association—apoplexy within hours of a discrete drinking episode—is the principal evidence in this case. The markedly elevated 24h urinary free cortisol at presentation, which fell within the reference range at 3-month follow-up, is most consistent with an acute, self-limited stress response at the onset of apoplexy compounded by glucose-driven osmotic diuresis, rather than autonomous hypercortisolism, and reinforces the importance of cautious interpretation of isolated extreme values.

### 4.4 Oculomotor nerve palsy in pituitary apoplexy

The oculomotor nerve is the cranial nerve most frequently affected in PA, owing to its course along the lateral wall of the cavernous sinus, where it is susceptible to compression by lateral tumor expansion and to raised intracavernous pressure; ischaemia of the nerve has also been proposed (14). Complete third-nerve palsy, as seen here, comprises ptosis, a dilated unreactive pupil and impaired adduction, elevation and depression. Importantly, isolated ophthalmoplegia without optic-pathway compromise carries a generally favourable prognosis, and recovery—whether after conservative or surgical management—is well documented, frequently over weeks to several months (14). Consistent with these reports, our patient’s complete third-nerve palsy showed partial recovery by 3 months, with reduced ptosis and improved ocular motility but a residual deficit, in line with the typically gradual time-course of recovery and supporting continued follow-up to document further improvement.

### 4.5 Surgical timing and endocrine considerations

The optimal timing of surgery in PA remains debated. A meta-analysis of 908 patients found no significant difference between early surgical and conservative strategies in the recovery of ophthalmoplegia, visual fields or visual acuity, supporting selective rather than reflexive early operation (15). Guideline-based practice reserves urgent decompression for progressive visual loss or declining consciousness, while clinically stable patients with preserved vision may be managed expectantly or semi-electively (9). In our patient, three considerations favored a short interval of stabilization before surgery: preserved visual fields, without clinically significant optic-pathway compromise, indicated the optic apparatus was not critically threatened; the isolated oculomotor palsy did not by itself mandate emergency decompression; and withholding aspirin allowed partial restoration of platelet function, reducing operative hemorrhagic risk in a patient with coronary stents. Throughout, the endocrine priority was unchanged—empirical stress-dose glucocorticoid cover preceded biochemical confirmation, reflecting that secondary adrenal insufficiency, not the tumor itself, poses the most immediate threat to life (3, 4).

Beyond the acute management of apoplexy, the care of pituitary tumors more broadly carries important neuropsychiatric and behavioral dimensions. Although our patient had a non-functioning SF-1 lineage gonadotroph PitNET treated surgically and therefore did not receive dopamine-agonist therapy, a systematic review and meta-analysis by Penchev et al. reported that dopamine-agonist treatment of prolactinomas can be associated with impulse control disorders (16). This contrast illustrates that therapeutic decisions in pituitary disease extend beyond tumor control to encompass quality-of-life and psychiatric considerations, reinforcing the value of individualized, multidisciplinary management across the spectrum of pituitary tumors.

### 4.6 Lessons from the present case

Four practical points emerge. First, acute headache with a cranial nerve palsy should prompt urgent sellar imaging and an assessment of corticotroph function, even without a known pituitary lesion. Second, in patients with cardiovascular risk factors, a discrete episode of heavy alcohol intake may act as a contributory trigger and warrants documentation, while recognizing the limits of causal inference. Third, WHO 2022 lineage-based classification—here an SF-1 gonadotroph PitNET with a low Ki-67 index—provides reproducible biological characterization and informs surveillance, since clinically non-functioning gonadotroph tumors typically present through mass effect (6, 17). Fourth, surgical timing should be individualized against visual status, antiplatelet considerations and endocrine stability rather than applied as a fixed rule. The 3-month outcome in this patient—gross total resection with no residual tumor on MRI, durable optic-pathway decompression, and partial recovery of the oculomotor palsy—supports this individualized approach, while the persistent multi-axis hypopituitarism underscores the need for lifelong endocrine replacement and surveillance. This report has limitations: the attribution to alcohol rests on temporal association alone and cannot exclude the patient’s hypertension, diabetes, antiplatelet therapy or spontaneous tumoral infarction as contributors; plasma ACTH and IGF-1 were not measured; GATA3 immunostaining was unavailable, and the extensive infarction restricted immunohistochemical evaluation to the residual viable tumor, which may account for the absence of detectable LH and estrogen receptor expression; a formal cardiology consultation was not obtained before antiplatelet interruption; and the levothyroxine dose remained low (12.5 µg daily) at the 3-month review, with stepwise up-titration planned at subsequent endocrine follow-up.

## 5 Conclusion

We report a PA temporally associated with acute alcohol intoxication, presenting with isolated complete oculomotor nerve palsy in a man with multiple cardiovascular risk factors, in whom histopathology confirmed an SF-1 lineage gonadotroph PitNET with extensive infarction. Immediate glucocorticoid cover and individually timed endoscopic transsphenoidal surgery after antiplatelet washout achieved gross total resection with an uncomplicated course, and at 3 months the patient showed no residual tumor, a decompressed optic apparatus and partial recovery of the oculomotor palsy, while remaining on stable replacement for persistent multi-axis hypopituitarism. The case underscores three messages relevant to endocrine practice: vigilance for apoplexy as an endocrine emergency when headache accompanies a cranial nerve palsy; the value of lineage-based PitNET classification; and the importance of individualized surgical timing and lifelong endocrine surveillance.

## Data Availability

The original contributions presented in the study are included in the article/**Supplementary Material**. Further inquiries can be directed to the corresponding author.

## Supplementary material

Supplementary Table 1 Representative reported cases of pituitary apoplexy precipitated by uncommon or non-classical factors, compared with the present case. Classical precipitants (major surgery, anticoagulation, dynamic pituitary testing, pregnancy) are well described and are not tabulated here; this comparison focuses on atypical triggers, of which alcohol is among the least reported. ACS, acute coronary syndrome; BEP, bleomycin-etoposide-cisplatin; CAD, coronary artery disease; CN III, oculomotor nerve; PitNET, pituitary neuroendocrine tumor; SF-1, steroidogenic factor-1; T2DM, type 2 diabetes mellitus. Dashes indicate data not specified in the source. The comparison illustrates that, in contrast to anticoagulation, hormonal stimulation, hypoperfusion and chemotherapy, a discrete episode of alcohol intake is rarely documented as a precipitant of pituitary apoplexy in the English-language literature.
