# Supplementary material for: Case Report: Pituitary apoplexy temporally associated with acute alcohol intoxication, presenting as complete oculomotor nerve palsy in an SF-1 lineage gonadotroph PitNET
Source: Front Endocrinol (Lausanne). 2026 Sep 24;17:1919724. doi: 10.3389/fendo.2026.1919724 (PMC13645506; doi:10.3389/fendo.2026.1919724)
Supplement: Supplementary file 1 [file SupplementaryFile1.pdf]

Supplementary Material

Case Report: Pituitary apoplexy temporally associated with acute alcohol intoxication, presenting as complete oculomotor nerve palsy in an SF-1 lineage gonadotroph PitNET — Article 1919724

Supplementary Table S1. Representative reported cases of pituitary apoplexy precipitated by uncommon or non-classical factors, compared with the present case. Classical precipitants (major surgery, anticoagulation, dynamic pituitary testing, pregnancy) are well described and are not tabulated here; this comparison focuses on atypical triggers, of which alcohol is among the least reported.

Study (year)	Age / Sex	Precipitant	Presentation	Management	Outcome
**Present case**	61 / M	Acute alcohol intake (binge) on a background of hypertension, T2DM, CAD on aspirin	Thunderclap headache; complete right oculomotor palsy; multi-axis hypopituitarism; SF-1 gonadotroph PitNET	Aspirin withdrawal; stress-dose hydrocortisone; semi-elective endoscopic transsphenoidal resection (day 8)	Gross total resection; partial CN III recovery and no residual tumour at 3 months; persistent hypopituitarism
Swaid et al. (2019)	Older adult / —	Anticoagulation (IV heparin + dual antiplatelet therapy for ACS)	Isolated unilateral oculomotor nerve palsy without visual-field defect	Conservative / supportive with endocrine cover	Reported recovery of oculomotor function
Andriuskeviciute et al. (2022), Front Endocrinol	40 / M	Anabolic androgenic steroid (exogenous testosterone) abuse	Abrupt headache; partial third-nerve palsy; haemorrhagic sellar lesion involving cavernous sinus	Endocrine work-up; individualised management	PA of a PitNET temporally associated with steroid use
Abo et al. (2023)	76 / M	Intraoperative hypovolaemia / blood loss during non-cardiac surgery	Postoperative visual and ophthalmic symptoms in a known macroadenoma	Recognition and endocrine management	Apoplexy attributed to hypoperfusion of the adenoma
Hamrick et al. (2022)	31 / M	Systemic chemotherapy (BEP regimen)	Headache, nausea, visual-field deficit one week after chemotherapy	Urgent transnasal resection	Hemorrhagic macroadenoma; review of prior chemotherapy-associated cases

ACS, acute coronary syndrome; BEP, bleomycin-etoposide-cisplatin; CAD, coronary artery disease; CN III, oculomotor nerve; PitNET, pituitary neuroendocrine tumor; SF-1, steroidogenic factor-1; T2DM, type 2 diabetes mellitus. Dashes indicate data not specified in the source. The comparison illustrates that, in contrast to anticoagulation, hormonal stimulation, hypoperfusion and chemotherapy, a discrete episode of alcohol intake is rarely documented as a precipitant of pituitary apoplexy in the English-language literature.
