# Supplementary material for: Case Report: Pituitary apoplexy temporally associated with acute alcohol intoxication, presenting as complete oculomotor nerve palsy in an SF-1 lineage gonadotroph PitNET
Source: Front Endocrinol (Lausanne). 2026 Sep 24;17:1919724. doi: 10.3389/fendo.2026.1919724 (PMC13645506; doi:10.3389/fendo.2026.1919724)
Supplement: Supplementary file 2 [file SupplementaryFile2.pdf]

CARE Checklist (2013) of information to include when writing a case report

Based on the official template at www.care-statement.org/checklist

Manuscript title: Case Report: Pituitary apoplexy temporally associated with acute alcohol intoxication, presenting as complete oculomotor nerve palsy in an SF-1 lineage gonadotroph PitNET

Journal: Frontiers in Endocrinology (Pituitary Endocrinology) | Corresponding author: Yunqian Li

Topic	Item No	Checklist Item Description	Reported on Page
**Title**	1	The diagnosis or intervention of primary focus followed by the words "case report".	Title ("Case Report: …")
**Key Words**	2	2 to 5 key words that identify diagnoses or interventions in this case report, including "case report".	Keywords
**Abstract**	3a	Introduction – What is unique about this case and what does it add to the scientific literature?	Abstract
	3b	Main symptoms and/or important clinical findings.	Abstract
	3c	The main diagnoses, therapeutic interventions, and outcomes.	Abstract
	3d	Conclusion – What is the main "take-away" lesson(s) from this case?	Abstract
**Introduction**	4	One or two paragraphs summarizing why this case is unique (may include references).	Sec. 1 (Introduction)
**Patient Information**	5a	De-identified patient specific information.	Sec. 2 (Case Description)
	5b	Primary concerns and symptoms of the patient.	Sec. 2
	5c	Medical, family and psychosocial history including relevant genetic information.	Sec. 2
	5d	Relevant past interventions with outcomes.	Sec. 2
**Clinical Findings**	6	Describe significant physical examination (PE) and important clinical findings.	Sec. 2
**Timeline**	7	Historical and current information from this episode of care organized as a timeline (figure or table).	Figure 3 (timeline)
**Diagnostic Assessment**	8a	Diagnostic testing (such as PE, laboratory testing, imaging, surveys).	Sec. 2; Table 1; Figs 1–2
	8b	Diagnostic challenges (such as access to testing, financial, or cultural).	Sec. 2 (UFC; ACTH/DST/GATA3 unavailable)
	8c	Diagnosis (including other diagnoses considered).	Sec. 2
	8d	Prognosis (such as staging in oncology) where applicable.	Sec. 2 (Ki-67); Sec. 4
**Therapeutic Intervention**	9a	Types of intervention (such as pharmacologic, surgical, preventive, self-care).	Sec. 2
	9b	Administration of intervention (such as dosage, strength, duration).	Sec. 2–3
	9c	Changes in intervention (with rationale).	Sec. 2–3
**Follow-up and Outcomes**	10a	Clinician- and patient-assessed outcomes (if available).	Sec. 3; Patient Perspective
	10b	Important follow-up diagnostic and other test results.	Sec. 3; Table 1; Fig 2
	10c	Intervention adherence and tolerability (How was this assessed?).	Sec. 3
	10d	Adverse and unanticipated events.	Sec. 2–3
**Discussion**	11a	A scientific discussion of the strengths AND limitations associated with this case report.	Sec. 4 (limitations paragraph)

Topic	Item No	Checklist Item Description	Reported on Page
	11b	Discussion of the relevant medical literature with references.	Sec. 4; Suppl. Table S1
	11c	The scientific rationale for any conclusions (including assessment of possible causes).	Sec. 4
	11d	The primary “take-away” lessons from this case report (without references) in a one paragraph conclusion.	Sec. 4 (four practical points); Conclusion
**Patient Perspective**	12	The patient should share their perspective on the treatment(s) they received.	Patient Perspective
**Informed Consent**	13	Did the patient give informed consent? Please provide if requested.	Ethics Statement — Yes

Section references map to manuscript sections; substitute final page numbers once the journal applies pagination. All 13 items (with subitems) are addressed in the manuscript.

Reference: Gagnier JJ, Kienle G, Altman DG, Moher D, Sox H, Riley D. The CARE guidelines: consensus-based clinical case report guideline development. J Clin Epidemiol. 2014;67:46–51.
